# Clinical application of indocyanine green fluorescence imaging navigation for pediatric renal cancer

**DOI:** 10.3389/fped.2023.1108997

**Published:** 2023-05-05

**Authors:** Jun Feng, Wei Yang, Hong Qin, Jiatong Xu, Shan Liu, Jianyu Han, Ning Li, Lejian He, Huanmin Wang

**Affiliations:** ^1^ Department of Surgical Oncology, Beijing Children’s Hospital, Capital Medical University, National Center for Children’s Health, Beijing, China; ^2^ Department of Pathology, Beijing Children’s Hospital, Capital Medical University, National Center for Children’s Health, Beijing, China; ^3^ Department of Surgical Urology, Beijing Children’s Hospital, Capital Medical University, National Center for Children’s Health, Beijing, China

**Keywords:** indocyanine green (ICG), fluorescence imaging, children, renal cancer, clinical application

## Abstract

**Background:**

Indocyanine Green (ICG) fluorescence imaging has been widely used in the surgical treatment of adult renal cancers, but its application in pediatric renal cancers has rarely been reported. This study aims to summarize the experience of ICG fluorescence imaging in pediatric renal cancers and explores its safety and feasibility.

**Methods:**

The clinical features, surgical information, ICG administration regimen, near infrared radiography data *in vivo* and ex vivo and pathological results of children with renal cancers using ICG navigation were analyzed and summarized.

**Results:**

There were 7 cases of renal cancer, including 4 cases of Wilms tumor (WT), 1 case of malignant rhabdoid tumor of the kidney (MRTK) and 2 cases of renal cell carcinoma (RCC). By intraoperative intravenous injection of ICG from 2.5 to 5 mg (0.05–0.67 mg/kg), the tumors were visualized in 6 cases *in vivo* or ex vivo, and the tumor visualization failed in 1 case due to renal artery embolization before operation. By injecting 5 mg ICG into the normal renal tissue during the operation, 3 patients achieved fluorescent localization of sentinel lymph nodes. No ICG-related adverse reactions were found in any of the patients during or after operation.

**Conclusions:**

ICG fluorescence imaging is safe and feasible for renal cancers in children. Intraoperative administration can achieve tumor and sentinel lymph node visualization which will facilitate the development of nephron sparing surgery (NSS). However, the technique is affected by ICG dose, anatomical conditions around the tumor, and renal blood flow. A proper dose of ICG and the complete removal of perirenal fat are helpful for the fluorescence imaging of the tumor. It has potential in the operation of renal cancer in children.

## 1. Introduction

Renal cancer is one of the most common malignant solid tumors in children, accounting for about 5% of all childhood cancers (1). At present, radical nephrectomy is still a gold standard for unilateral renal tumors, but more and more studies advocate nephron sparing surgery (NSS) to retain more normal renal tissue and reduce the loss of renal function and the risk of end-stage renal disease on the long-term (2, 3). However, the biggest challenge of NSS is how to completely remove the tumor and make the margin negative. Therefore, how to effectively distinguish the tumor from normal renal tissue by strengthening the recognition of the tumor during operation is the key to avoiding residue and retaining more renal function.

In the surgical treatment of adult renal cell carcinoma (RCC), the most commonly used tumor recognition technique is near-infrared fluorescence imaging based on indocyanine green (ICG) (4). Due to the different uptake of ICG between the tumor and the normal renal tissue, when ICG enters the kidney through blood circulation, they show different fluorescence types that hypofluorescence or afluorescence in the tumor and hyperfluorescence in the normal kidney. This enhances the intraoperative recognition of the tumor tissue boundary and preserves more renal tissue while ensuring the negative margin (5). At present, ICG navigation has been widely used in partial nephrectomy of adult RCC, and may become a routine step. Besides, ICG navigation also has been used in other solid tumors in adults, such as liver cancer, breast cancer, colon cancer and prostate cancer, as well as for identifying tumors, metastatic lesions and sentinel lymph nodes in real time during operation (6–10). However, for children, ICG navigation has been more used in liver tumors, but rarely used in other solid tumors (11–18).

In this study, we hypothesized that renal cancers in children can also have different ICG uptake from normal kidney, just like adult renal carcer, showing different fluorescence types. We performed ICG fluorescence imaging in children with renal cancers during radical nephrectomy to evaluate the safety and feasibility of ICG navigation, and to gain the experience of identifying renal cancers, delineating tumor boundaries and locating sentinel lymph nodes in children.

## 2. Materials and methods

### 2.1. Patients

Seven children with renal cancers who underwent ICG navigation surgery in the Department of Surgical Oncology at Beijing Children's Hospital affiliated to Capital Medical University from January 2022 to July 2022 were analyzed retrospectively. The clinical features, surgical information, ICG administration regimen, near infrared imaging data *in vivo* and ex vivo and pathological results of the 7 cases were collected and summarized. ICG navigation surgery has obtained the informed consent of all the guardians of the children, and this study has been approved by the Ethics Committee of Beijing Children's Hospital [(2022)-E-167-R].

### 2.2 ICG navigation

All the children underwent radical nephrectomy by open surgery and were given ICG during the operation. Twenty-five mg of sterile indocyanine green (Dandong Yichuang Pharmaceutical Co., China) was dissolved in 10 ml sterile distilled water (2.5 mg/ml) to prepare the ICG solution. After the removal of Gerota's fascia and perirenal fat, 2.5–5 mg of ICG (0.05–0.67 mg/kg) was injected intravenously, and images of the tumor and kidney were taken using real-time near infrared photography. The fluorescence types of the tumor and the normal kidney were observed, the fluorescence intensity was measured, and the tumor boundary was outlined according to the fluorescence difference.

After radical nephrectomy, we routinely removed all lymph nodes around the renal vessels and suspected lymph nodes between the inferior vena cava and the abdominal aorta. During the operation, ICG was used to localize the sentinel lymph nodes of the kidney in 3 cases. Before ligating renal arteries and veins, 5 mg of ICG was injected into normal renal tissue, and then near-infrared photography was taken around the renal vessels to find and remove fluorescent lymph nodes.

After injection of ICG, we observed whether the child had fever, rash and shock symptoms associated with allergic reactions. Postoperative complications were evaluated according to Clavien-Dindo classification (19).

We used two open fluorescence camera systems (FLI-10B, Nanjing Nuoyuan Medical Devices CO., Ltd; OPTO-CAM2100, Guangdong Optomedic Technologies, Inc.) to perform ICG navigation surgery, which also have the function of measuring fluorescence intensity.

By measuring the fluorescence intensity, the fluorescence difference between the tumor and the normal kidney was quantitatively analyzed. The fluorescence intensity of the normal kidney was taken as background in order to eliminate the effects of the ICG dose, time and individual differences in ICG metabolism on fluorescence intensity, and the tumor-background ratio (TBR) was calculated.

### 2.3 Data analysis and statistics

SPSS 22.0 statistical software was used to analyze and process the data. The continuous variable of the normal distribution is expressed by the mean ± standard deviation, and the continuous variable of the non-normal distribution is expressed by the median (lower quartile, upper quartile).

## 3. Results

### 3.1. Clinical features

**Figure 1:**
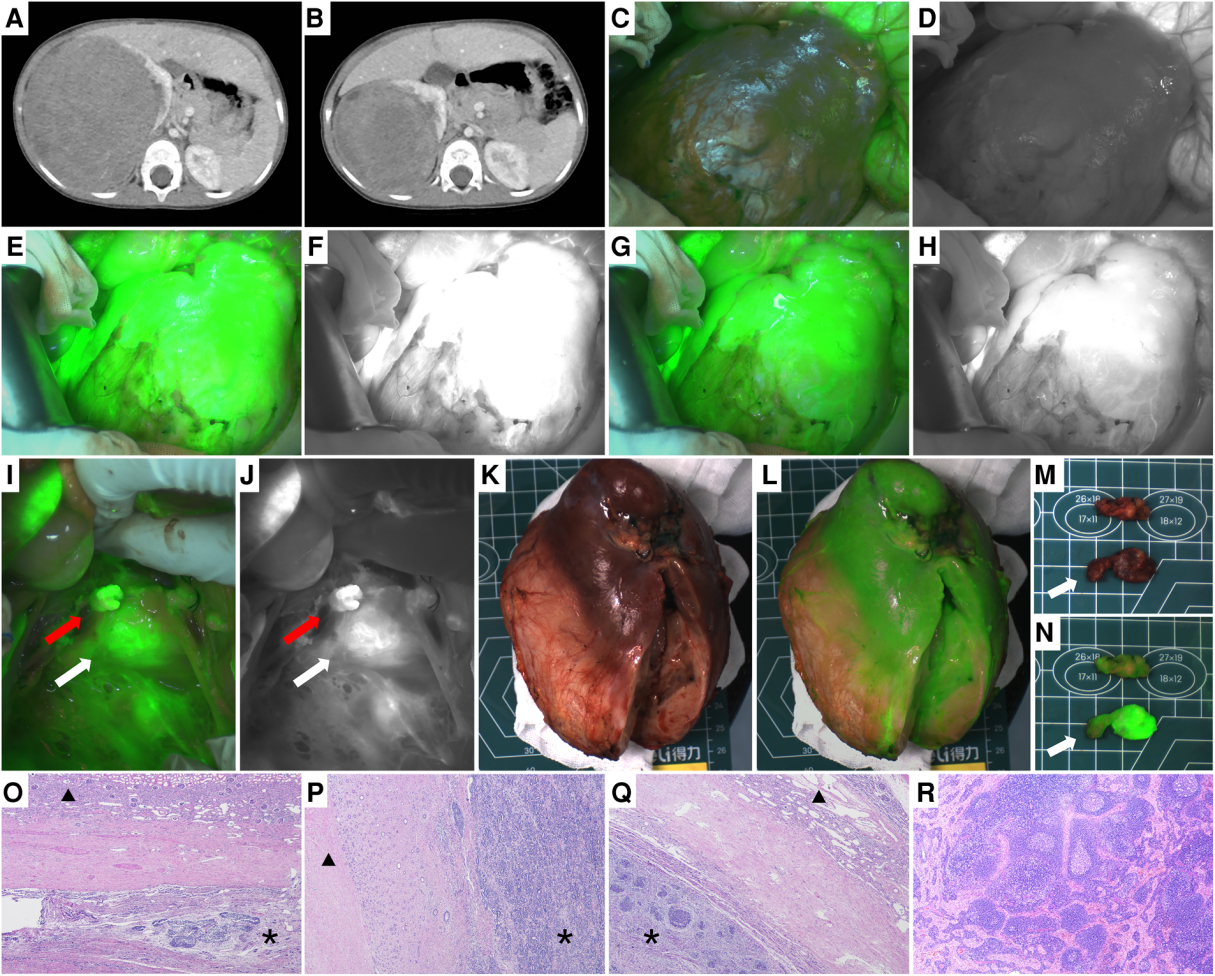
Case 2, the image of the right renal tumor before and after chemotherapy, intraoperative and ex vivo near infrared photography. (**A,B**) CT images of the right renal tumors before and after chemotherapy. (**C,D**) Images of the right kidney and tumor before ICG injection. (**E,F**) Fifteen seconds after injection of ICG, both the normal kidney and tumor showed hyperfluorescence. (**G,H**) Three minutes after injection of ICG, the fluorescence difference between the normal kidney and tumor was the most obvious, and the boundary between them could be seen clearly. (**I,J**) Fluorescence images of renal vessels and surrounding lymph nodes. The red arrow points to the ligated renal vessels and the white arrow points to the lymph nodes of ICG (+). (**K,L**) White light and fluorescence images of the right kidney and tumor ex vivo. (**M,N**) White light and fluorescence images of ICG (+) lymph nodes ex vivo, with white arrows pointing to ICG (+) lymph nodes. (**O–Q**) Histopathological images of the boundary between the tumor and normal renal tissue (H&E, 40×). * Indicates the tumor and ▴ represents the normal renal tissue. There are clear boundaries between the two kinds of tissues. (**R**) Histopathological image of ICG (+) lymph nodes that were not invaded by the tumor.

**Figure 2:**
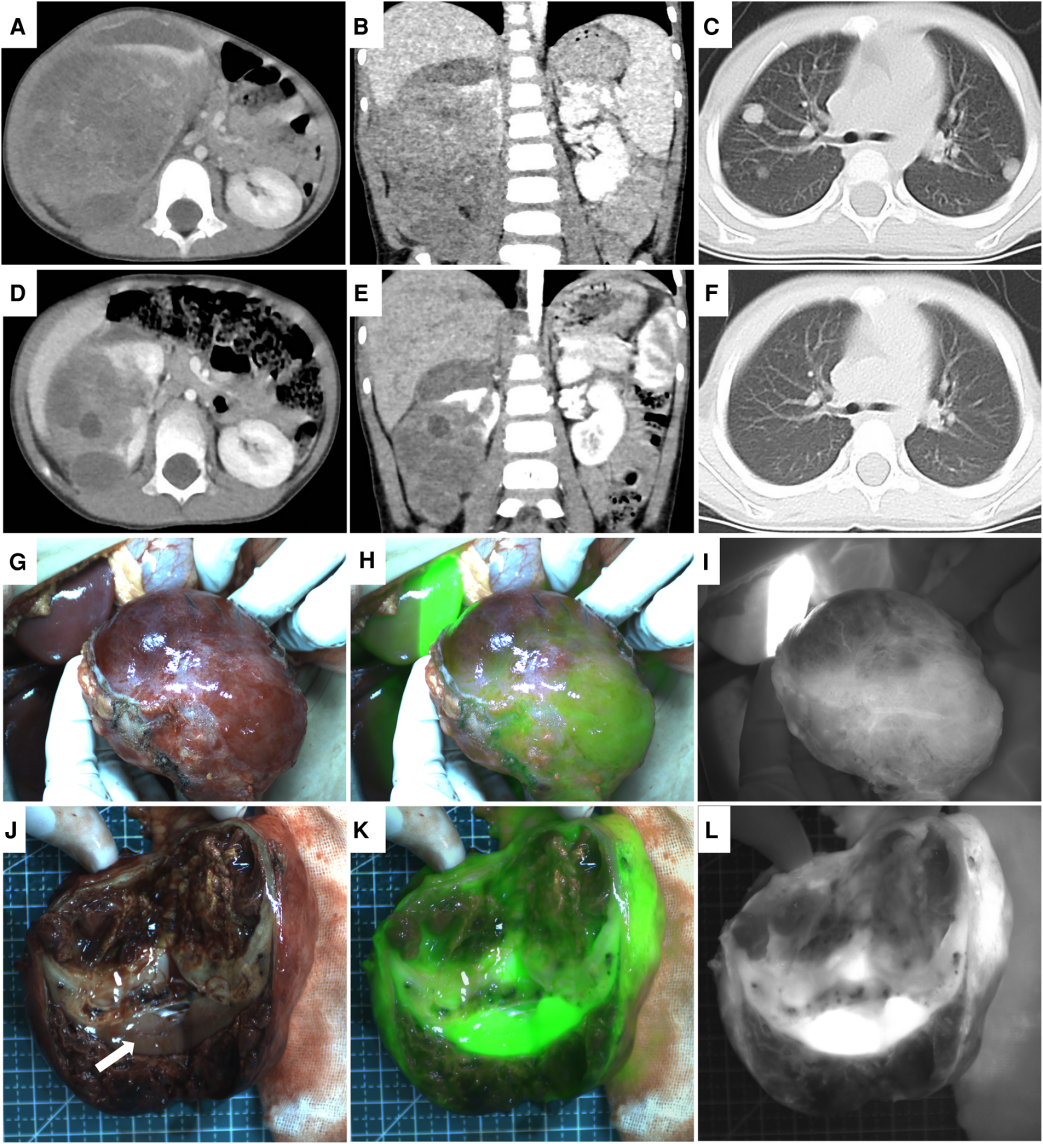
Case 3, pre- and post-chemotherapy imaging, intraoperative and ex vivo near infrared photography of the tumor. (**A–C**) CT images of the right renal tumor and lung metastases before chemotherapy. (**D–F**) CT images of the right renal tumor and lung metastases after chemotherapy showed hematoma around the tumor. (**G–I**) White light and fluorescence images of the right kidney and tumor during operation. The tumor adhered to the surrounding tissue. Twenty seconds after injection of ICG, the right kidney and tumor showed diffuse fluorescence imaging. (**J–L**) White light and fluorescence images of the right kidney and tumor ex vivo. The white arrow points to the normal renal tissue, showing hyperfluorescence. The tumor with fibrosis and necrosis can be seen above the normal renal tissue, showing hypofluorescence. Hematoma can only be seen under normal renal tissue, showing afluorescence.

**Figure 3:**
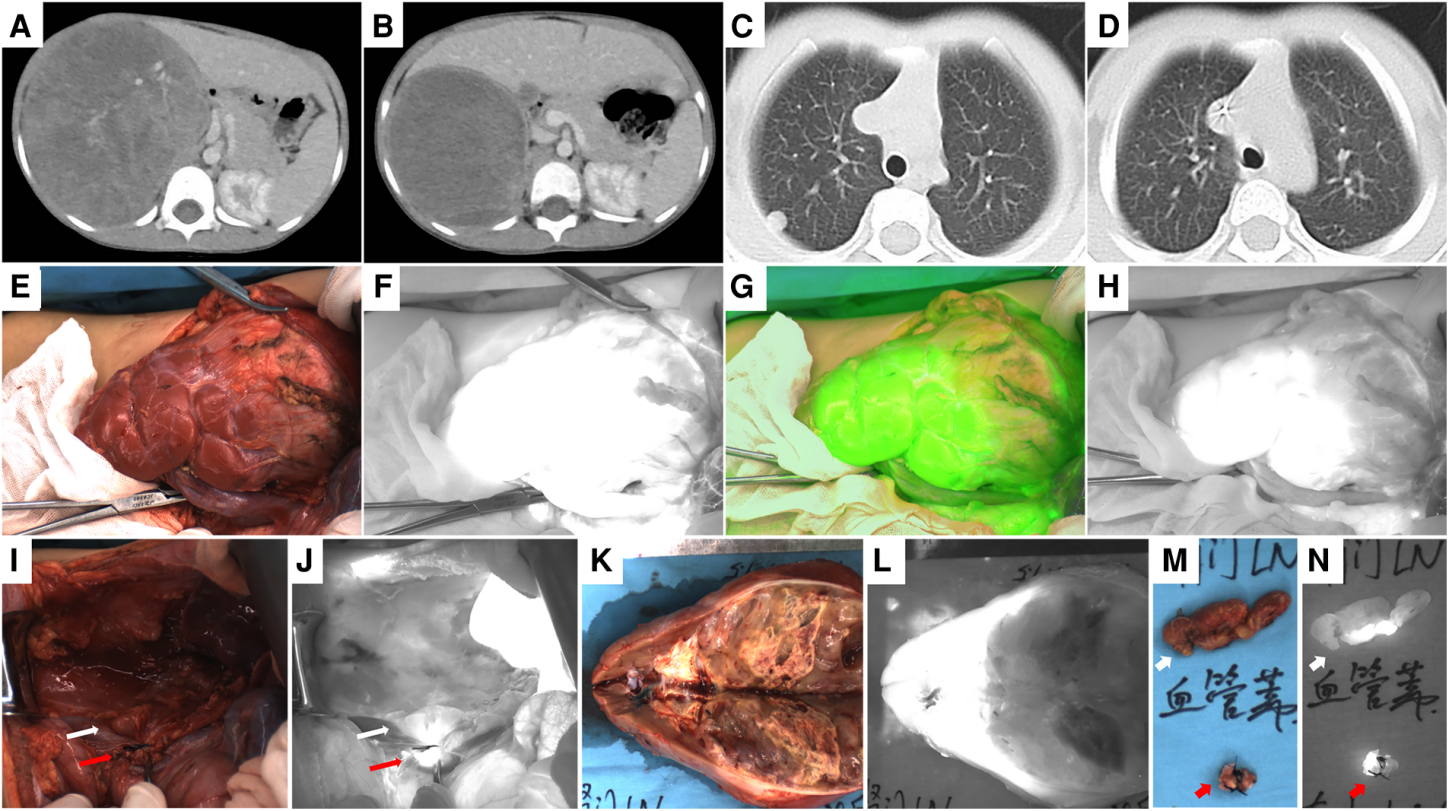
Case 7, pre-and post-chemotherapy images, intraoperative and ex vivo near infrared photography of the tumor. (**A,B**) CT images of the right renal tumor before and after chemotherapy. (**C,D**) CT images of lung metastases before and after chemotherapy. (**E,F**) Fifteen seconds after ICG injection, both the normal kidney and tumor showed hyperfluorescence. (**G,H**) Seven minutes after ICG injection, the fluorescence difference between the normal kidney and the tumor is the most obvious, and the boundary between them can be seen clearly. (**I,J**) White light and fluorescence images of the renal vessels and surrounding lymph nodes. The red arrow points to the ligated renal vessels and the white arrow points to the lymph nodes of ICG (+). (**K,L**) White light and fluorescence images of the right kidney and the tumor ex vivo. (**M,N**) White light and fluorescence images of renal vessels and ICG (+) lymph nodes ex vivo, with red arrows pointing to renal vessels and white arrows pointing to ICG (+) lymph nodes.

A total of 7 patients were enrolled in this study, with 3 males and 4 females. The median age was 49 (22, 101) months. Among them, 4 patients were diagnosed with Wilms tumor (WT), 1 patient was diagnosed with malignant rhabdoid tumor of the kidney (MRTK), and 2 patients were diagnosed with RCC. 3 patients with WT and 1 with RCC received preoperative chemotherapy (Table 1). Because of the tumor size, lung metastasis and renal vein tumor thrombus with lung metastasis respectively, cases 2, 3 and 7 (WT) had preoperative chemotherapy according to SIOP. After that, the tumor in case 2 shrank significantly (Figures 1A,B); in case 3, the tumor shrank and most of the lung metastases disappeared (Figures 2A–F); and in case 7, the tumor shrank, the renal vein tumor thrombus disappeared, and the lung metastasis shrank (Figures 3A–D). Case 5 (RCC) was firstly diagnosed with WT because of fever, hematuria and imaging examination showing a right renal tumor, and right renal vein and inferior vena cava tumor thrombus. As a result, he received chemotherapy, but it was found that there was no significant reduction in the tumor size. Therefore, the patient was transferred to Beijing Children's Hospital. Eventually, he was diagnosed with RCC through an ultrasound-guided tumor biopsy and pathology. The tumor thrombus of the inferior vena cava grew near the atrium. In order to reduce intraoperative bleeding, the right renal artery embolization was performed before the operation.

**Table 1:** Clinical characteristics and information of ICG navigation surgery in 7 cases with renal cancers.

NO.	Sex	Age (months)	Preoperative diagnosis	Preoperative chemotherapy	Preoperative examination	Weight (kg)	ICG dosage	Maximum diameter of tumor before surgery (cm)	Tumor location	Time from intravenous injection of ICG to the appearance of fluorescence	Time from intravenous injection of ICG to tumor visualization	Fluorescence pattern of tumor	TBR	Pathology of tumor	Tumor staging	Postoperative chemotherapy	Follow-up (months)	Prognosis
1	F	11	MRTK	NA	CT/US/MRI	7.5	5 mg (0.67 mg/kg)	2.5	Left	10 s	5 min	Hyperfluorescent	1.4	MRTK	IV	UH-I	7	Alive
2	F	29	WT	VA	CT/US	14	2.5 mg (0.18 mg/kg)	11	Right	15 s	3 min	Hypofluorescent	0.5	WT (Mixed)	SIOP II (Intermediate risk)	VAD	5	Alive
3	F	49	WT	VAD	CT/US	14	2.5 mg (0.18 mg/kg)	9.7	Right	20 s	NA	Hypofluorescent (ex vivo)	0.3 (ex vivo)	WT (Mixed)	SIOP IV (High risk)	VAD	5	Alive
4	M	15	WT	NA	CT/US	12	2.5 mg (0.21 mg/kg)	8.4	Right	15 s	1 min	Hypofluorescent	0.4	WT(Cystic partially differentiated)	SIOP I (Low risk)	EE4A	2	Alive
5	M	154	Xp11.2 tRCC	VAD	CT/US	52	2.5 mg (0.05 mg/kg)	10.7	Right	NA	NA	NA	NA	Xp11.2 tRCC	IV	NA	2	Alive
6	M	138	Xp11.2 tRCC	NA	CT/US	32	2.5 mg (0.08 mg/kg)	16.3	Left	20 s	NA	Hypofluorescent (ex vivo)	0.3 (ex vivo)	Xp11.2 tRCC	IV	NA	1	Alive
7	F	64	WT	VAD	CT/US	15.5	2.5 mg (0.16 mg/kg)	9.9	Right	15 s	7 min	Hypofluorescent	0.6	WT(Mixed)	SIOP IV (High risk)	VAD	1	Alive

ICG, Indocyanine Green; TBR, Tumor-Background Ratio; MRTK, Malignant Rhabdoid Tumor of the Kidney; WT, Wilms Tumor; Xp11.2 tRCC, renal cell carcinoma associated with Xp11.2 translocation/TFE3 gene fusion; VA, vincristine/dactinomycin; VAD, vincristine/dactinomycin/doxorubicin; UH-1, vincristine/doxorubicin/cyclophosphamide/etoposide/carboplatin; EE4A, vincristine and dactinomycin for 18 weeks; SIOP, International Society of Paediatric Oncology; CT, Computed Tomography; US, Ultrasound; MRI, Magnetic Resonance Imaging.

**Figure 4:**
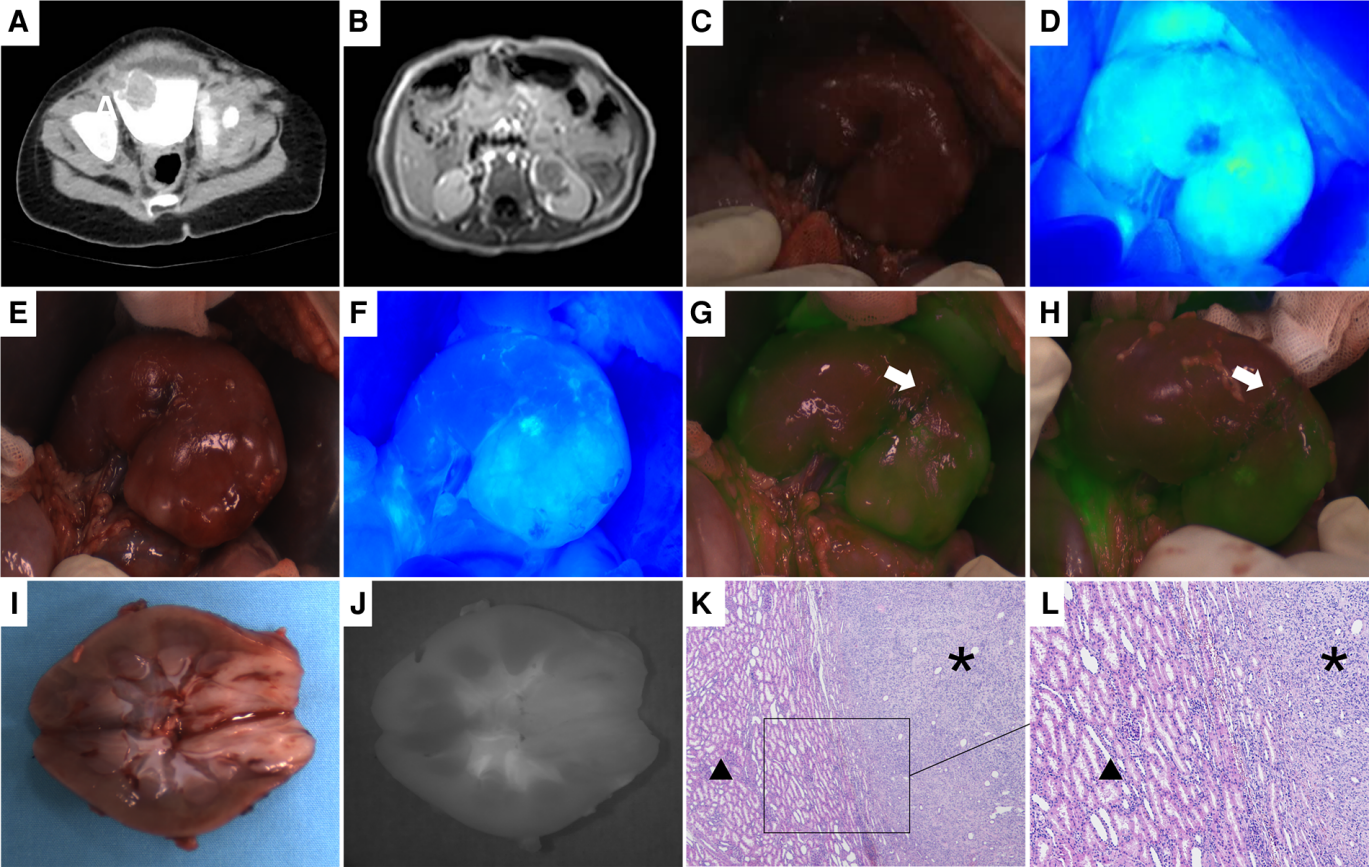
Case 1, the preoperative image of the tumor, intraoperative and ex vivo near infrared photography. (**A**) CT image of bladder tumor. (**B**) MRI image of the left renal tumor. (**C,D**) White light and fluorescence images of the left kidney. Ten seconds after ICG injection, the left kidney and tumor showed hyperfluorescent. (**E,F**) Five minutes after ICG injection, the tumor still showed hyperfluorescence, while the normal kidney showed hypofluorescence, and a clear boundary could be seen between them. (**G,H**) The cutting line was drawn according to the fluorescence difference between the tumor and the normal kidney, and the white arrow points to the cutting line. (**I,J**) White light and fluorescence images of the left kidney and tumor ex vivo. (**K,L**) Histopathological images of the boundary between the tumor and normal renal tissue (H&E, 40×; H&E, 100×). * Indicates the tumor and ▴ represents the normal renal tissue. There are clear boundaries between the two kinds of tissues.

**Figure 5:**
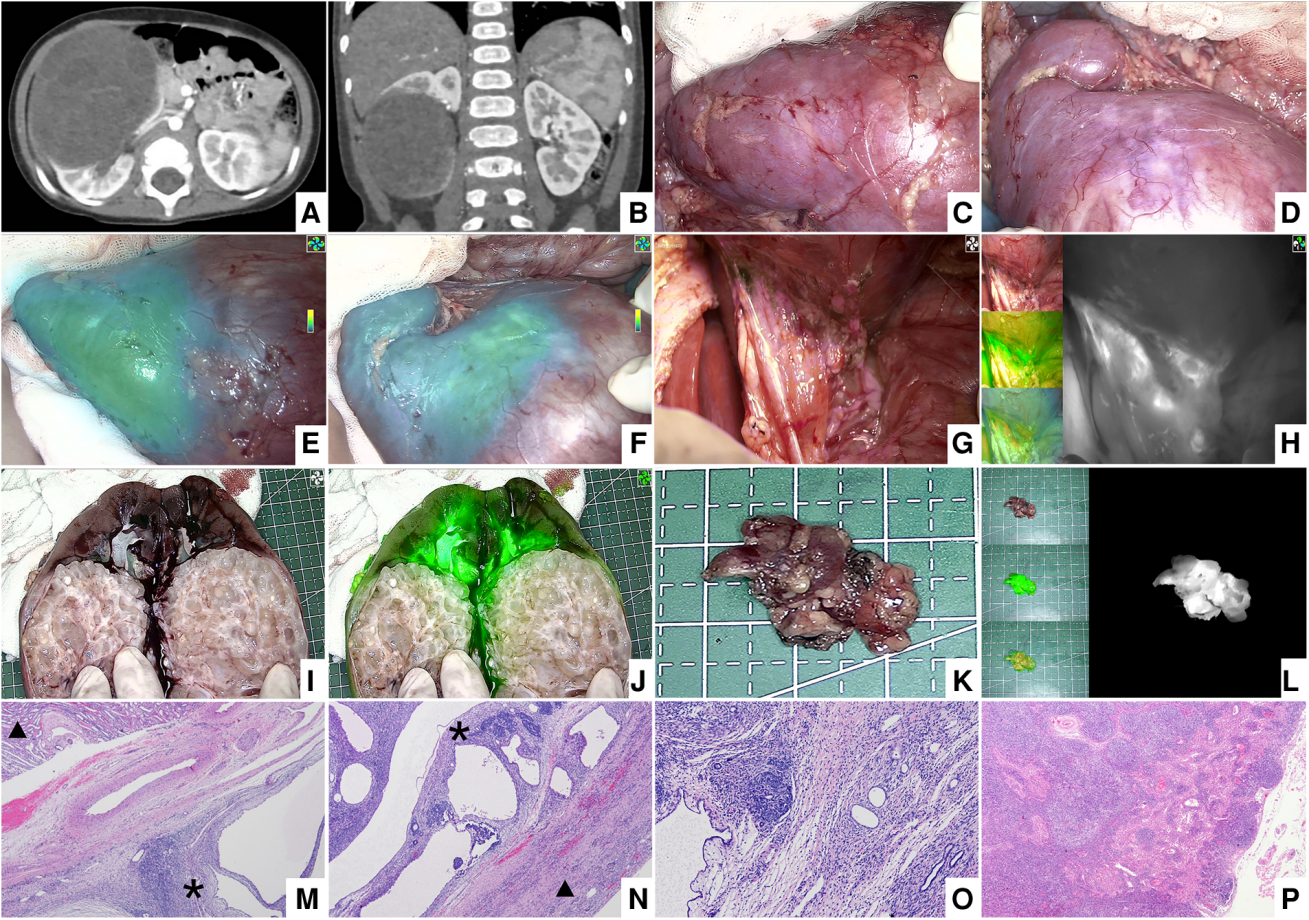
Case 4, the preoperative image of the right renal tumor, intraoperative and ex vivo near infrared photography. (**A,B**) CT images of the right renal tumor before operation. (**C–F**) White light and fluorescence images of the right kidney and tumor after injection of ICG. (**G,H**) White light and fluorescence images of the lymph nodes around the renal vessels. (**I,J**) White light and fluorescence images of the right kidney and tumor ex vivo. (**K,L**) White light and fluorescence images of ICG (+) lymph nodes ex vivo. (**M,N**) Histopathological images of the boundary between the tumor and normal renal tissue (H&E, 40×). * Indicates the tumor and ▴ represents the normal renal tissue. There are clear boundaries between the two kinds of tissues. (**O**) Histopathological image of tumor (H&E, 100×). (**P**) Histopathological image of ICG (+) lymph nodes that were not invaded by the tumor.

**Figure 6:**
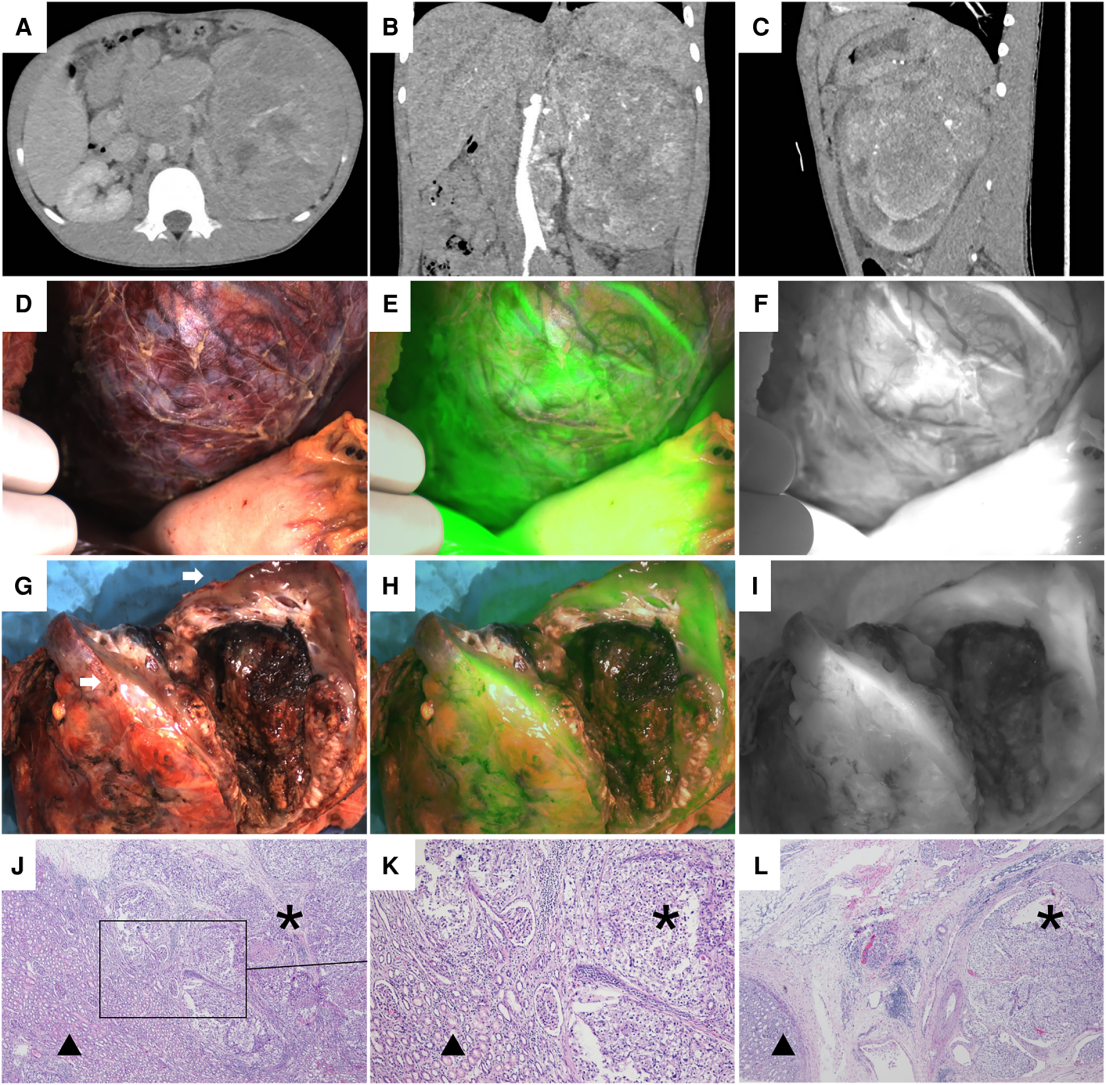
Case 6, the preoperative image of the left renal tumor, intraoperative and ex vivo near infrared photography. (**A–C**) CT images of the left renal tumor before operation. (**D–F**) White light and fluorescence images of the left kidney and tumor during operation. The tumor adhered to the surrounding tissue. Twenty seconds after ICG injection, the left kidney and tumor showed diffuse fluorescence imaging. (**G–I**) White light and fluorescence images of the left kidney and tumor ex vivo. The white arrow points to the normal kidney tissue, showing hyperfluorescence. The tumor can only be seen under the normal renal tissue, showing afluorescence. (**J–L**) Histopathological images of the boundary between the tumor and normal renal tissue (H&E, 40×; H&E, 100×; H&E, 40×). * Indicates the tumor and ▴ represents the normal renal tissue. There are clear boundaries between the two kinds of tissues.

Cases 1, 4, and 6 did not receive chemotherapy before operation. Case 1 showed a bladder tumor and a left kidney tumor in the CT and MRI because of hematuria (Figures 4A,B). In order to confirm the pathological diagnosis, a urologist performed a tumor resection through cystoscope, and the pathology results showed a malignant rhabdoid tumor. After a comprehensive evaluation, no other lesions were found except the left renal tumor, so radical nephrectomy was performed. A right renal tumor was found in case 4 because of an abdominal mass. He was diagnosed as WT according to the imaging findings (Figures 5A,B). There was no metastasis or venous tumor thrombus, and radical nephrectomy was performed. In case 6, the left renal tumor was found because of abdominal pain. According to the imaging findings, the left renal tumor was diagnosed as RCC without metastasis, but there were enlarged retroperitoneal lymph nodes and left renal vein tumor thrombus protruding to the inferior vena cava (Figures 6A–C). Surgical resection of the tumor and venous tumor thrombus was selected.

### 3.2. ICG navigation surgery and fluorescence imaging

Unilateral radical nephrectomy and lymph node dissection were performed in 7 cases, 2 cases on the left side and 5 cases on the right side. The median diameter of the tumors before operation was 9.9 (9.05, 10.85) cm. In 2 cases of RCC, venous tumor thrombus and adrenal gland invaded by the tumor were also removed. Table 2 shows the histopathological results of 7 cases.

**Table 2:** Histopathological results of 7 cases of renal cancers.

NO.	Histological classification	Renal capsule	Renal sinus	Renal pelvis	Ureter	Tumour thrombus	Perirenal fat	Lymph nodes	Necrosis
1	MRTK	(−)	(−)	Invaded	(−)	(−)	(−)	0/6	0
2	WT (Mixed)	(−)	(−)	(−)	(−)	(−)	(−)	0/5	5%
3	WT (Mixed)	(−)	(−)	Invaded	(−)	(−)	(−)	0/11	80%
4	WT (Cystic partially differentiated)	(−)	(−)	Invaded	(−)	(−)	(−)	0/3	0
5	Xp11.2 tRCC	(−)	Invaded	Invaded	(−)	renal vein and vena cava inferior	(−)	0/68	80%
6	Xp11.2 tRCC	(−)	Invaded	(−)	(−)	renal vein and vena cava inferior	Involved	3/16	30%
7	WT (Mixed)	(−)	Invaded	(−)	(−)	(−)	(−)	0/23	20%

MRTK, Malignant Rhabdoid Tumor of the Kidney; WT, Wilms Tumor; Xp11.2 tRCC, renal cell carcinoma associated with Xp11.2 translocation/TFE3 gene fusion.

“(−)” means it is not invaded by tumor.

4 cases of renal tumors (cases 1, 2, 4, and 7), including 3 cases of WT and 1 case of MRTK, had real-time tumor visualization during operation through intraoperative administration successfully. In these 4 cases, Gerota's fascia was cut open, perirenal fat was removed, 2.5 to 5 mg of ICG (0.16–0.67 mg/kg) was injected through peripheral vein, and near-infrared photography of tumor and normal kidney was taken at the same time. The tumor and the normal kidney showed fluorescence 10–15 s later (Figures 1C–F, 3E,F, 4C,D). After 1–7 min of administration, there was a significant difference in fluorescence between the tumor and the normal kidney (Figures 1G,H, 3G,H, 4E,F, 5C–F). Because of this, the operator could identify the tumor and label the tumor boundary according to the fluorescence difference (Figures 4G,H). Then, combined with white light observation and palpation, it was confirmed that the fluorescence boundary was consistent with the actual tumor boundary, and the fluorescence difference between tumor and normal kidney still existed until ex vivo (Figures 1K,L, 3K,L, 4I,J, 5I,J). It is worth noting that the fluorescence of WT and MRTK were completely opposite. The fluorescence of WT showed hyperfluorescence in the normal kidney and hypofluorescence or afluorescence in the tumor (Figures 1, 3, 5), while the fluorescence of MRTK showed hyperfluorescence in the tumor and hypofluorescence in the normal kidney (Figure 4). The TBR values of the 3 cases of WT *in vivo* were 0.5,0.4, and 0.6 respectively. The TBR value of 1 case of MRTK *in vivo* was 1.4.

Case 3 (WT) and case 6 (RCC) also showed fluorescence in the tumor and the normal kidney after intraoperative administration. However, due to severe adhesion between the tumor and the hematoma, perirenal fat, and Gerota's fascia in case 3 and 6 (Figures 2G–I, 6D–F), the tumor and its boundary could not be accurately identified, and the tumor could not be visualized during operation. However, when the tumor was dissected ex vivo, a significant difference in fluorescence was observed between the tumor and the normal renal tissue. In both cases, the fluorescence of the normal renal tissue was hyperfluorescent, while the tumor was hypofluorescent or afluorescent (Figures 2J–L, 6G–I). The TBR values ex vivo were all 0.3.

Case 5 (RCC) underwent right renal artery embolization before operation, and severe adhesion between the tumor and the surrounding tissue was found during the operation. Only the diffuse fluorescence of the retroperitoneal tissue was observed after administration, and afluorescence was found in the tumor and the normal renal tissue after dissection of the tumor ex vivo.

In 3 cases of WT (cases 2, 4, and 7), 5 mg of ICG was injected into the normal renal tissue before ligation of the renal vessels, and 5 min later, ICG (+) lymph nodes were seen around the renal vessels. The fluorescent localization of sentinel lymph nodes was achieved (Figures 1I,J,M,N, 3I,J,M,N, 5G,H,K,L). These sentinel lymph nodes were resected under the guidance of ICG fluorescence, and no tumor was found by pathology (Figures 1R, 5P).

### 3.3. The relationship between fluorescence types and histopathology

Except for case 5, the fluorescence of the other 6 tumors was significantly different from that of normal renal parenchyma *in vivo* or ex vivo, and the difference and boundary between the two kinds of tissues could also be seen under microscope (Figures 1O–Q, 4K,L, 5M–O, 6J–L). Table 3 showed the fluorescence types and TBR values of different pathological types of tumors. Both WT and RCC showed hyperfluorescence in normal kidney and hypofluorescence or afluorescence in tumor (TBR < 1). MRTK showed hyperfluorescence in tumor and hypofluorescence in normal kidney, and the TBR value > 1.

**Table 3:** Relationship between fluorescence types and histopathology of renal cancers.

Histopathology	Tumor	Normal renal parenchyma	TBR
MRTK (*n* = 1)	Hyperfluorescent	Hypofluorescent or afluorescent	1.4
WT (*n* = 4)	Hypofluorescent or afluorescent	Hyperfluorescent	0.45 (0.38, 0.53)
Xp11.2 tRCC (*n* = 1*)	Hypofluorescent or afluorescent	Hyperfluorescent	0.3

MRTK, Malignant Rhabdoid Tumor of the Kidney; WT, Wilms Tumor; Xp11.2 tRCC, renal cell carcinoma associated with Xp11.2 translocation/TFE3 gene fusion; TBR, Tumor-Background Ratio.

* There was no fluorescence perfusion in tumor and normal renal parenchyma in 1 RCC child due to renal artery embolization before surgery.

### 3.4. Follow-up and prognosis

All the children had no fever, allergy, infection, and hepatic and renal insufficiency before operation. After the injection of ICG, we did not find fever, rash or shock symptoms associated with allergic reactions in any case until the end of the operation. According to the Clavien-Dindo classification of postoperative complications, case 4 and 7 were defined as grade I and only needed analgesia, antiemetic, and electrolyte supplementation. Cases 5 and 6 were transferred to intensive care unit after removal of tumor thrombus in the inferior vena cava and were defined as grade IV. Case 5 also had thrombus in the inferior vena cava and was given anticoagulation therapy. The other 3 cases were defined as grade II, and all of them had elevated levels of AST (84.2–208 U) and ALT (51.1–109.5 U) after operation (the normal ranges of AST and ALT were 14–44 U/L and 7–30 U/L, respectively). We consider that the main reasons were surgical trauma and traction of the liver during the operation. AST and ALT returned to normal levels after 3 to 5 days of drug treatment. Case 1 and 6 were diagnosed as stage I acute renal injury due to elevated creatinine after operation, which was considered to be related to nephrectomy. They were all discharged from the hospital 6–10 days after operation and continued to receive treatment and follow-up. All of them survived healthily. The median follow-up time was 2 (1.5, 5) months.

## 4. Discussion

With the continuous improvement of treatment strategy, the success rate of renal cancer treatment in children is improving, among which the cure rate of WT has reached more than 90% (1). In addition to tumor treatment, the focus of research in recent years has shifted to the long-term quality of life of children, in which the protection of renal function is very important (2). Therefore, more and more surgeons perform NSS on children with unilateral WT. Studies have found that NSS is safe for children with low stage or low risk, has the same cancer control effect as radical nephrectomy, and can reduce the risk of postoperative renal insufficiency (3, 20, 21). However, the implementation of NSS is also faced with difficult technical challenges, while retaining more nephron, not only to ensure that the margin is negative, but also to avoid tumor rupture during operation. It requires the operator to accurately identify the tumor and the normal renal tissue during the operation. Therefore, it is necessary to find an effective imaging technique to visualize the tumor during the operation, rather than relying on the operator's experience to determine the location of the tumor.

At a technical level of intraoperative imaging of renal cancer, we have learned a lot through experiences with adult RCC. In recent years, ICG fluorescence imaging has been the most commonly used technique to identify RCC. ICG is one of the few fluorescent dyes approved by the Food and Drug Administration (FDA) that can be used in humans. It has no nephrotoxicity and allergic reactions are rare. ICG can emit fluorescence under the excitation of near-infrared light with a wavelength of 700–850 nm (14, 22). When ICG enters the kidney with blood flow, due to the presence of bilirubin transferase transporting ICG in the proximal and distal renal tubules, the normal renal tissue shows hyperfluorescence and lasts for a period of time, while the cancerous tissue lacks the expression of this enzyme, and all tumors show hypofluorescence or afluorescence (23, 24).

Through this technique, RCC and normal renal tissue show significantly different fluorescence, so that the operator can identify the tumor and its boundary, and complete partial nephrectomy while ensuring the negative margin. Therefore, ICG navigation has been widely used in partial nephrectomy of adult RCC. However, this technique is rarely used in renal cancer surgery in children (13–18). We applied this technique to renal cancers in children and reported our initial experience.

In our study, 6 cases had tumors which could be distinguished from the normal kidney according to the difference in fluorescence intensity *in vivo* or ex vivo. 4 cases of WT and 1 case of RCC showed hyperfluorescence in the normal kidney and hypofluorescence or afluorescence in the tumor (Table 3), which was consistent with the fluorescence type of WT and adult RCC (23, 25, 26). The fluorescence of MRTK was contrary to that of WT and RCC. The tumor showed hyperfluorescence, while the normal kidney showed hypofluorescence, suggesting that ICG accumulated more in the tumor. Our study is the first report on ICG fluorescence imaging of MRTK. Although the specific mechanism is not clear, we speculate that MRTK may have the enhanced permeability and retention (EPR) effect like many adult or child malignant solid tumors. Therefore, the accumulation of ICG in tumor tissues increased (14, 27–29). The quantitative calculation of fluorescence intensity also made up for the subjective judgment, and the results suggest that there may be a potential relationship between fluorescence types and pathology, which will be the direction of our further research. In the report of adult renal tumors, compared with the normal kidney, RCC and chromophobe cell carcinoma showed hypofluorescence or afluorescence, oncocytoma showed isofluorescence, while benign cysts showed isofluorescence or hyperfluorescence. The sensitivity and specificity of hypofluorescence in the malignant lesions were 84% and 57%. Although the fluorescence type could not reliably predict the benign or malignant tumors, it can still provide supplementary information about the tumor during the operation (23, 26, 30). Although our number of cases is limited, it includes the common types of pediatric renal cancers, which has accumulated experience for further understanding the fluorescence imaging of pediatric renal cancers.

In this study, 4 cases (cases 1, 2, 4 and 7), including 3 cases of WT and 1 case of MRTK, we were able to observe an obvious difference in fluorescence intensity and a clear boundary between the tumor and the normal kidney during operation, so that we could outline the boundary of the tumor. The TBR values of these 4 cases also confirmed the difference of fluorescence intensity. In cases 3(WT) and 6(RCC), due to the adhesion of the tumor to perirenal fat and Gerota's fascia, it was difficult to distinguish the fluorescence type between the tumor and the normal kidney during operation. However, after dissection ex vivo, it could still be seen that there was a significant difference in fluorescence intensity and a clear boundary between them. In the histopathological images of these 6 cases, we can also see that there is a clear boundary between tumor and normal renal tissue, which is the histological basis for using fluorescence imaging to depict the tumor boundary. Therefore, according to the results of this study, it is feasible to identify renal cancers and normal kidney in children by ICG navigation. Although all children in this study underwent radical nephrectomy, our initial experience provides a research basis for ICG navigation NSS in the future.

However, the fluorescence imaging of renal cancers based on ICG is also affected by many factors. First, the dose and time of ICG will affect the fluorescence imaging of tumor. Although ICG navigation has been successfully applied in adult partial nephrectomy, there is still no standardized ICG administration regimen. The dose, concentration and frequency were quite different, although the drugs were administered through the peripheral vein after the tumor and kidney were exposed during operation (5, 23, 26, 31, 32). Angell et al. (30) explored a model of ICG administration. They injected a low dose of ICG, tested the degree of fluorescence difference between the tumor and the normal kidney, and then calibrated the dose according to the fluorescence difference, so as to obtain the best fluorescence difference. In this way, the efficiency can reach up to 86%. Our study also found that after intraoperative administration, the tumor and normal renal tissue showed fluorescence, and the boundary became blurred. It was necessary to wait for part of the ICG to be absorbed by hepatocytes, and then the boundary between the tumor and the normal renal tissue could be identified clearly. In the meantime, the retroperitoneal tissue also showed fluorescence after administration, which affected the observation of the tumor and the normal kidney. Therefore, this study explores the feasibility of using ICG in identifying renal cancers in children. To the further directions of the study, it may be necessary to reduce the dose, and the appropriate ICG administration will be great helpful to developing the use of NSS in children.

Secondly, the anatomical condition around the tumor is also an influencing factor. At present, most of the fluorescence devices widely used can only implement the fluorescence imaging in near-infrared window I (NIR-I, 700–900 nm), and the maximum depth of detecting fluorescent dyes is 1 cm (33). Therefore, it will be very difficult for the operator to identify the tumor, due to the attenuation of fluorescence imaging caused by the deep location of the tumor or the adhesion of too much perirenal fat to the surface of the tumor (31, 32, 34). However, compared with the NIR-1 window, second NIR window (NIR-II, 1,000–1,700 nm) has better detection depth and contrast (35, 36). ICG, as a type of NIR-II fluorescent dye, has been used in intraoperative NIR-II fluorescence imaging of liver cancer, and achieved a higher tumor detection rate (37). Therefore, NIR-II fluorescence imaging technique is expected to be used in the surgery of renal cancer in children. In cases 3 and 6, because of the adhesion between the tumor and the surrounding tissue, we couldn't identify the fluorescence type of the tumor and the normal kidney during the operation. Furthermore, the perirenal fat as retroperitoneal tissue also emitted fluorescence, resulting in diffuse fluorescence development in the operation area. However, there was no adhesion of perirenal fat in cases 1, 2, 4 and 7, so the tumor and the normal kidney could be directly observed after the perirenal fat was removed, which made it easy to identify. In order to obtain better fluorescence imaging, perirenal fat should be fully removed during the operation. ICG navigation surgery may not have advantages for patients with tumor adhesion to perirenal tissue.

Third, ICG navigation is based on different degrees of ICG uptake by the tumor and the normal kidney, which cannot be carried out when the renal blood supply system is blocked. Therefore, this technique may not suitable for patients who need a renal artery embolization before operation to reduce intraoperative bleeding.

In this study, the fluorescence localization of sentinel lymph nodes was performed in 3 cases of WT. The results were satisfactory and the sentinel lymph nodes were labeled successfully. In recent years, in order to improve the therapeutic effect of renal tumors in children, both COG and SIOP suggested that a sufficient number of lymph nodes should be collected during operation (38, 39). Pachl (40) and Abdelhafeez et al. (41) also obtained the lymph node localization using intraparenchymal and perihilar injection of ICG. Therefore, this method is feasible. This is due to the fact that the renal cortex is rich in lymphoid tissue and returns to the lymphatics around the renal vessels (42). Therefore, when ICG enters the lymphatics of the renal cortex, it can locate the sentinel lymph nodes along the lymphatic reflux. However, the lymphatic reflux of normal renal parenchyma may not be the same as that of tumors, so we are not sure that there is a certain relationship between the sentinel lymph nodes of ICG (+) and tumor lymphatic reflux in this study, and further studies are needed to confirm the reliability of this method. The perihilar injection used by Abdelhafeez et al. (41) can overcome this problem by injecting ICG into the lymphatic pathway shared by tumor and normal renal parenchyma, but the effect of fluorescence localization is not as good as intraparenchymal injection, and in their study, the sample size is also very small, so the final conclusion can not be reached yet. Although there is no tumor metastasis in these ICG (+) lymph nodes, this fluorescence localization technique makes it possible to visualize the sentinel lymph nodes, which provides the possibility for accurate sentinel lymph node resection and adequate lymph node sampling in the future.

Although the application of ICG navigation in children with renal cancers is limited by many factors, this technique is convenient to use, which makes it possible to visualize renal cancers and sentinel lymph nodes, and makes it easier for operators to determine the boundary of the tumor. It will be a useful technique to assist NSS and sentinel lymph node resection.

There are still limitations in this study. As a single-center retrospective study, the ability of ICG navigation to identify renal tumors in children has not been fully studied due to the small number of cases. The effectiveness of the method of locating sentinel lymph nodes in this study also needs to be verified by more cases. Moreover, since this study is our initial experience, the intraoperative dose of ICG is still in the exploratory stage. Therefore, it is necessary to expand the sample size, carry out prospective research and multicenter cooperation.

## 5. Conclusion

The application of ICG navigation in children with renal cancers is safe and feasible. The intraoperative administration of ICG can visualize the renal cancers during operation, and make it easy for the operator to identify the tumor and outline the boundary of the tumor. It is expected that this technique will be used in NSS of pediatric renal cancers. However, the dose, anatomical conditions around the tumor and renal blood flow will affect the tumor fluorescence imaging. Using a proper dose of ICG and complete removal of perirenal fat are helpful to the fluorescence imaging of the tumor and locating the sentinel lymph nodes. It has potential in the operation of renal cancer in children.

## Data Availability

The original contributions presented in the study are included in the article, further inquiries can be directed to the corresponding authors.
